# Effectiveness of CONUT and NRI as nutritional risk screening tools in peritoneal dialysis: a multicenter study

**DOI:** 10.3389/fnut.2025.1544338

**Published:** 2025-05-09

**Authors:** Wenlong Qiu, Rui Chu, Yaqi Ma, Jiaxin Xue, Qingdong Xu, Yueqiang Wen, Xiaojiang Zhan, Fenfen Peng, Xiaoyang Wang, Juan Wu, Ning Su, Xiaoran Feng, Xingming Tang, Xianfeng Wu, Qian Zhou, Na Tian

**Affiliations:** ^1^Department of Nephrology, General Hospital of Ningxia Medical University, Yinchuan, China; ^2^Ningxia Clinical Research Center of Kidney Disease, Yinchuan, China; ^3^Department of Nephrology, Jiangmen Central Hospital, Jiangmen, China; ^4^Department of Nephrology, The Second Affiliated Hospital of Guangzhou Medical University, Guangzhou, China; ^5^Department of Nephrology, The First Affiliated Hospital of Nanchang University, Nanchang, China; ^6^Department of Nephrology, Zhujiang Hospital, Southern Medical University, Guangzhou, China; ^7^Department of Nephrology, The First Affiliated Hospital of Zhengzhou University, Zhengzhou, China; ^8^Department of Nephrology, The First Affiliated Hospital, Sun Yat-sen University, Guangzhou, China; ^9^Department of Hematology, The Sixth Affiliated Hospital of Sun Yat-Sen University, Guangzhou, China; ^10^Department of Nephrology, Jiujiang No. 1 People’s Hospital, Jiujiang, China; ^11^Department of Nephrology, DongGuan SongShan Lake Tungwah Hospital, Dongguan, China; ^12^Department of Nephrology, Affiliated Sixth People’s Hospital, Shanghai Jiao Tong University, Shanghai, China; ^13^Department of Medical Statistics, Clinical Trials Unit, The First Affiliated Hospital, Sun Yat-sen University, Guangzhou, China

**Keywords:** peritoneal dialysis, nutritional risk screening, controlling nutritional status score, nutritional risk index, GLIM criteria, all-cause mortality, multicenter study

## Abstract

**Background:**

Nutritional risk is a significant concern for patients undergoing peritoneal dialysis (PD), adversely affecting their quality of life and increasing the risk of infections and complications. Effective screening tools are needed to identify high-risk patients for targeted interventions. This study investigates whether different nutritional assessment methods, like the Controlling Nutritional Status (CONUT) score and Nutritional Risk Index (NRI), correlate with patient prognosis, highlighting the importance of selecting appropriate screening tools to improve clinical outcomes in PD patients.

**Methods:**

This multicenter retrospective cohort study initially collected data from 2,427 patients across 10 centers, but ultimately included a cohort of 2,105 PD patients to evaluate the prevalence of malnutrition assessed using both the CONUT and NRI and its independent effects on all-cause mortality. Statistical analyses included log-rank tests, Cox regression models and the receiver operating characteristic curves to evaluate the association between nutritional risk and mortality.

**Results:**

Our findings revealed that 76.58% of patients were classified as having nutritional risk according to the CONUT score, while 79.10% by the NRI. Patients with nutritional risk exhibited a significantly higher all-cause mortality rate (log-rank test, *p* < 0.001). Cox regression analysis demonstrated that severe nutritional risk was an independent predictor of all-cause mortality, with adjusted hazard ratios of 2.55 (95% CI, 1.34–4.85; *p* = 0.007) for the CONUT score and 2.64 (95% CI, 1.74–4.03; *p* < 0.001) for the NRI. Kaplan–Meier survival curves highlighted the correlation between nutritional risk and survival.

**Conclusion:**

CONUT and NRI are effective for initial nutritional risk screening in PD patients, enabling clinicians to identify risk individuals who should undergo diagnostic assessments for a more comprehensive nutritional evaluation. Their simplicity and ease of implementation support integration into routine practice, making it feasible for healthcare providers to conduct regular screenings. Future studies should validate dynamic monitoring approaches.

## Introduction

Nutritional risk is a prevalent complication among patients undergoing peritoneal dialysis (PD), significantly impacting their quality of life and overall health outcomes. The increasing utilization of PD as a replacement therapy for chronic kidney disease has underscored the urgent need to address nutritional risk stratification within this population. Despite recognizing this issue, there is a lack of standardized screening protocols in clinical practice.

While the Global Leadership Initiative on Malnutrition (GLIM) criteria provide a two-step diagnostic framework (screening followed by phenotypic/etiologic confirmation), traditional methods like the Subjective Global Assessment (SGA) require multidisciplinary expertise and advanced measurements (e.g., muscle mass quantification), limiting their feasibility in routine practice (1, 2). However, patients undergoing PD require routine nutritional risk assessments, making it vital to have simplified tools for initial screening. The Controlling Nutritional Status (CONUT) score and Nutritional Risk Index (NRI) offer simplified and cost-effective alternatives, utilizing readily available clinical data. While these tools have been widely used in assessing nutritional status in other diseases (3, 4), their application in PD patients remains underexplored.

This multicenter study aims to evaluate the utility of CONUT and NRI as nutritional risk screening tools in PD patients. First, we assessed the prevalence of different nutritional risk at baseline using both scores. Second, we examined their independent associations with long-term all-cause mortality, adjusting for confounders such as inflammation and residual renal function. Finally, we propose a stepped care model: CONUT/NRI for initial risk stratification followed by GLIM-based confirmation for high-risk cases. Through this approach, we aim to standardize nutritional risk monitoring in PD care, enabling timely interventions that may mitigate morbidity and mortality. Our findings underscore the importance of integrating simplified screening tools into routine practice while emphasizing the need for confirmatory diagnostics to address the multifactorial nature of malnutrition.

## Materials and methods

### Study populations

**Figure 1:**
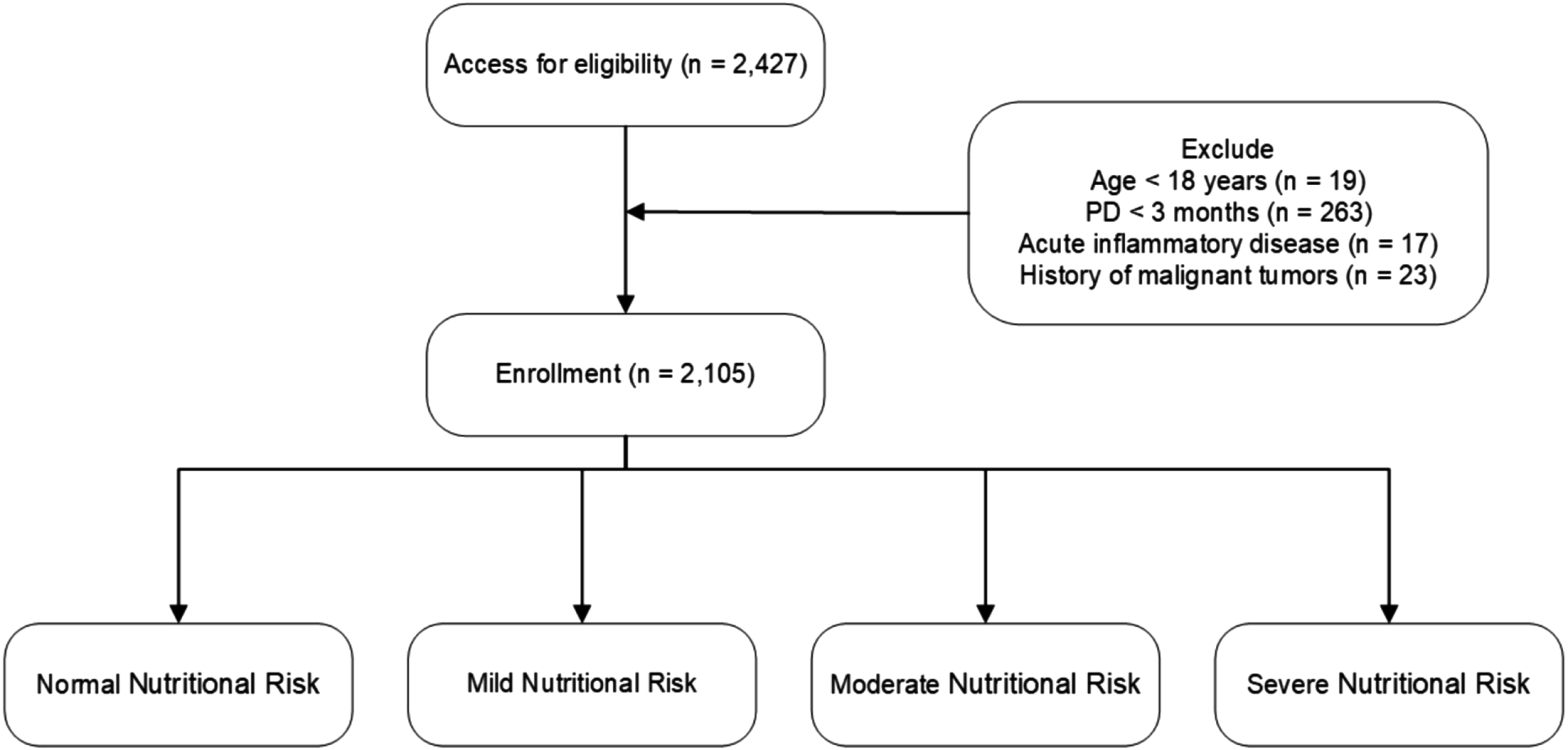
Flowchart of participant enrollment and exclusion criteria. Nutritional risk categories are defined as follows: CONUT score—Normal (0–1), Mild (2–4), Moderate (5–8), Severe (9–12); NRI—Normal (≥100), Mild (97.50–99.99), Moderate (83.50–97.49), Severe (<83.50).

This multicenter, observational cohort study enrolled 2,427 patients on peritoneal dialysis (PD) from 10 Chinese peritoneal dialysis centers between March 1, 2005, and February 28, 2023. The study included patients who started PD and were at least 18 years old, with a minimum duration of PD of 3 months. Patients were excluded if they had acute inflammatory disease during the baseline period or a history of malignant tumors. Ultimately, 2,105 patients were included and followed up until May 31, 2023, or until reaching an endpoint (death, kidney transplantation, transfer to hemodialysis, transfer to other centers, or loss to follow-up) (Figure 1). All patients provided informed consent. This study was conducted in accordance with the ethical standards of the Declaration of Helsinki and its amendments, with approval from the Human Ethics Committee.

### Baseline investigations

Baseline demographic and clinical information was collected at the commencement of PD therapy. All baseline demographic and clinical data were collected within the first 3 months after PD initiation. The baseline data included demographic information, medical comorbidities, medications, and laboratory indicators obtained from electronic medical records. Demographic variables consisted of age, sex, body mass index (BMI), and medical history related to diabetes (ICD-10 E10-E14), hypertension (I10-I15), hyperlipidemia (E78), and cardiovascular disease (CVD). CVD was defined as documented coronary artery disease (I20-I25), heart failure (I50), cerebrovascular disease (I60-I69), or peripheral artery disease (I70-I79), with all diagnoses based on the International Classification of Diseases, Tenth Revision (ICD-10) codes and corroborated by physician assessment from medical records. Laboratory indicators included leukocyte count, hemoglobin levels, serum albumin, creatinine, blood urea nitrogen, triglycerides, total cholesterol, serum phosphorus, serum calcium, and serum potassium. These laboratory metrics were evaluated using standard measurement techniques employed by each PD center’s laboratory.

Medical histories were recorded according to the initial page of the patients’ medical records, while additional data were gathered from hospitalization records and physicians’ orders. Clinicians at each PD center reviewed the patients’ electronic medical records, and trained researchers entered the data into a database, which was subsequently verified by trained graduate students. Patients attended follow-up assessments at their respective centers every 1 to 3 months, and trained nurses conducted monthly phone interviews to monitor their overall health status.

### Nutritional risk screening tools

Given the characteristic progression of malnutrition in patients with peritoneal dialysis (PD) due to factors such as inadequate nutrient intake, protein losses through dialysis, and chronic inflammation (5), we selected both the Controlling Nutritional Status (CONUT) score and the Nutritional Risk Index (NRI) to screen for nutritional risk in these patients.

The CONUT score is calculated based on serum albumin, total cholesterol, and lymphocyte count (6). The scoring ranges are categorized as normal (0–1), mild (2–4), moderate (5–8), or severe nutritional risk (9–12).

The NRI is calculated using the formula (7):


$$\begin{array}{l} NRI=\left(1.519\times \mathrm{serum albumin}\;\left(g/L\right)\right)\\ \;+\left(41.7\times \left[\frac{\mathrm{current body weight}\;\left(\mathrm{kg}\right)}{\mathrm{usual body weight}\;\left(\mathrm{kg}\right)}\right]\right) \end{array}$$

For our study, the usual body weight was replaced by the ideal body weight, following previous studies and using the Lorenz formulas:

For males:


$$\mathrm{Ideal body weight}\;\left(\mathrm{kg}\right)=\mathrm{height}\;\left(\mathrm{cm}\right)-100-\left[\frac{\mathrm{height}\;\left(\mathrm{cm}\right)-150}{4}\right]$$

For females:


$$\mathrm{Ideal body weight}\;\left(\mathrm{kg}\right)=\mathrm{height}\;\left(\mathrm{cm}\right)-100-\left[\frac{\mathrm{height}\;\left(\mathrm{cm}\right)-150}{2.5}\right]$$

When the current weight exceeds the ideal weight, we set the weight ratio as


$$\frac{\mathrm{ideal weight}}{\mathrm{current weight}}=1$$

We classified patients into four nutritional risk categories based on the established NRI grading criteria: severe nutritional risk (NRI < 83.5), moderate (83.50 ≤ NRI < 97.49), mild (97.50 ≤ NRI < 100), and normal (NRI ≥ 100).

### Outcome

The main outcome assessed was all-cause mortality. Each patient was monitored until one of the following events occurred: death, transition to hemodialysis, kidney transplantation, referral to other medical facilities, loss to follow-up, or until the end of the study on May 31, 2023.

### Statistical analysis

All statistical analyses were performed by SPSS (version 25.0) and R (version R-4.4.1). All tests were two-sided, and *p* < 0.05 was considered statistically significant. The Kolmogorov–Smirnov normality test was used to determine whether variables conformed to a normal distribution. Continuous variables that conformed to a normal distribution were expressed as the mean ± standard deviation (SD), and non-normally distributed variables are expressed as the median and interquartile range (IQR). Categorical variables are expressed in terms of numbers and percentage (n, %).

Univariate and multivariate Cox regression analyses were performed to evaluate hazard ratios (HRs) and 95% confidence intervals (CIs) of significant risk predictors based on overall survival. Kaplan–Meier curves and log-rank tests were used to present time-to-event data and compare survival between groups, respectively. Time-area under the curve (AUC) were calculated to assess and compare the discrimination capacity of the three malnutrition indexes to predict mortality. For the subgroup analysis, patients were stratified by gender, age, and body mass index (BMI) to assess the impact of these variables on all-cause mortality. To test whether the pattern of association varied across stratifications, we estimated multiplicative interactions by including the product term (exposure × stratification variable) in the models.

## Results

### Baseline characteristics of the patients

The final sample consisted of 2,105 patients who met the study’s inclusion and exclusion criteria. At a mean follow-up of 94.62 months, 328 cases of deaths were recorded (Figure 1). The median albumin level was 36.00 g/L (35.79 ± 6.16 g/L), A significant portion of the PD patients had comorbid conditions: 23.09% of patients had diabetes, 77.67% had hypertension, 13.78% had a history of cardiovascular disease (CVD) events, and 10.21% had hyperlipidemia. Additional baseline characteristics data for the study population were detailed in Table 1.

**Table 1:** Demographic and laboratory values of the study population.

Variables	Total (*n* = 2,105)
Age, Mean ± SD, years	51.07 ± 14.54
Gender, *n* (%)	
Male	1,125 (53.44)
Female	980 (46.56)
PD Vintage, Mean ± SD, Months	94.62 ± 45.55
BMI, M (Q₁, Q₃), kg/m^2^	21.63 (19.67, 24.05)
Smoking History, *n* (%)	
No	2011 (95.53)
Yes	94 (4.47)
Drinking History, *n* (%)	
No	2077 (98.67)
Yes	28 (1.33)
Diabetes, *n* (%)	
No	1,619 (76.91)
Yes	486 (23.09)
Hypertension, *n* (%)	
No	470 (22.33)
Yes	1,635 (77.67)
CVD History, *n* (%)	
No	1815 (86.22)
Yes	290 (13.78)
Hyperlipidemia, *n* (%)	
No	1890 (89.79)
Yes	215 (10.21)
Total Kt/V, M (Q₁, Q₃)	2.21 (1.79, 2.71)
Serum Albumin, Mean ± SD, Months, g/L	35.79 ± 6.16
RRF, M (Q₁, Q₃), ml/min	3.54 (1.93, 6.45)
WBC, M (Q₁, Q₃), 10^9^/L	6.31 (5.00, 7.78)
RBC, M (Q₁, Q₃), 10^12^/L	3.16 (2.63, 3.80)
Hemoglobin, M (Q₁, Q₃), g/L	91.00 (76.00, 109.00)
FBG, M (Q₁, Q₃), mmol/L	4.70 (4.11, 5.60)
Serum Creatinine, M (Q₁, Q₃), μmol/L	743.00 (568.00, 966.00)
Calcium, M (Q₁, Q₃), mmol/L	2.13 (1.95, 2.30)
Phosphorus, M (Q₁, Q₃), mmol/L	1.65 (1.33, 2.01)
iPTH, M (Q₁, Q₃), pg./ml	190.50 (74.20, 355.00)
Total Cholesterol, M (Q₁, Q₃), mmol/L	4.50 (3.66–5.40)
Triglycerides, M (Q₁, Q₃), mmol/L	1.37 (0.96, 1.95)
Nutrition Risk	
Any degree of nutrition risk, *n* (%)	
COUNT	1,612 (76.58)
NRI	1,665 (79.10)

SD: standard deviation, M: Median, Q₁: 1st Quartile, Q₃: 3rd Quartile. BMI, body mass index; CVD, cardiovascular disease; RRF, residual renal function; WBC, white blood cell; RBC, red blood cell; FBG, fasting blood glucose; iPTH, intact parathyroid hormone.

### Prevalence of nutritional risk

The prevalence of nutritional risk in the study population was 79.10% using the CONUT and 76.58% by used the NRI (Table 1) Notably, 67.7% of patients were classified as moderate-to-severe risk by CONUT (53.59% moderate, 14.11% severe), whereas 75.82% fell into mild-to-moderate risk categories by NRI (47.08% mild, 28.74% moderate) (Table 2). These discrepancies underscore the tool dependent variability in risk stratification.

**Table 2:** Prevalence of nutritional risk according to two different scoring systems.

Nutritional indices		Nutritional Risk			
		Normal	Mild	Moderate	Severe
CONUT, points		(0–1)	(2–4)	(5–8)	(9–12)
Formula	Albumin, g/dl (score)	≥3.5 (0)	3.0–3.4 (2)	2.5–2.9 (4)	<2.5 (6)
	Total cholesterol, mmol/L (score)	≥180 (0)	140–199 (1)	100–139 (2)	<100 (3)
	Lymphocyte count, × 10^9^/L (score)	≥1.60 (0)	1.20–1.59 (1)	0.80–1.19 (2)	<0.80 (3)
Study population, *n* (%)		493 (23.42%)	187 (8.88%)	1,128 (53.59%)	297 (14.11%)
NRI, points		≥100	97.50–99.99	83.50–97.49	<83.50
Formula	1.519 albumin (g/L) + 41.7 [current body weight [kg]/ideal weight (kg)]				
Study population, *n* (%)		440 (20.9%)	991 (47.08%)	605 (28.74%)	69 (3.28%)

### Nutritional risk and mortality

**Figure 2:**
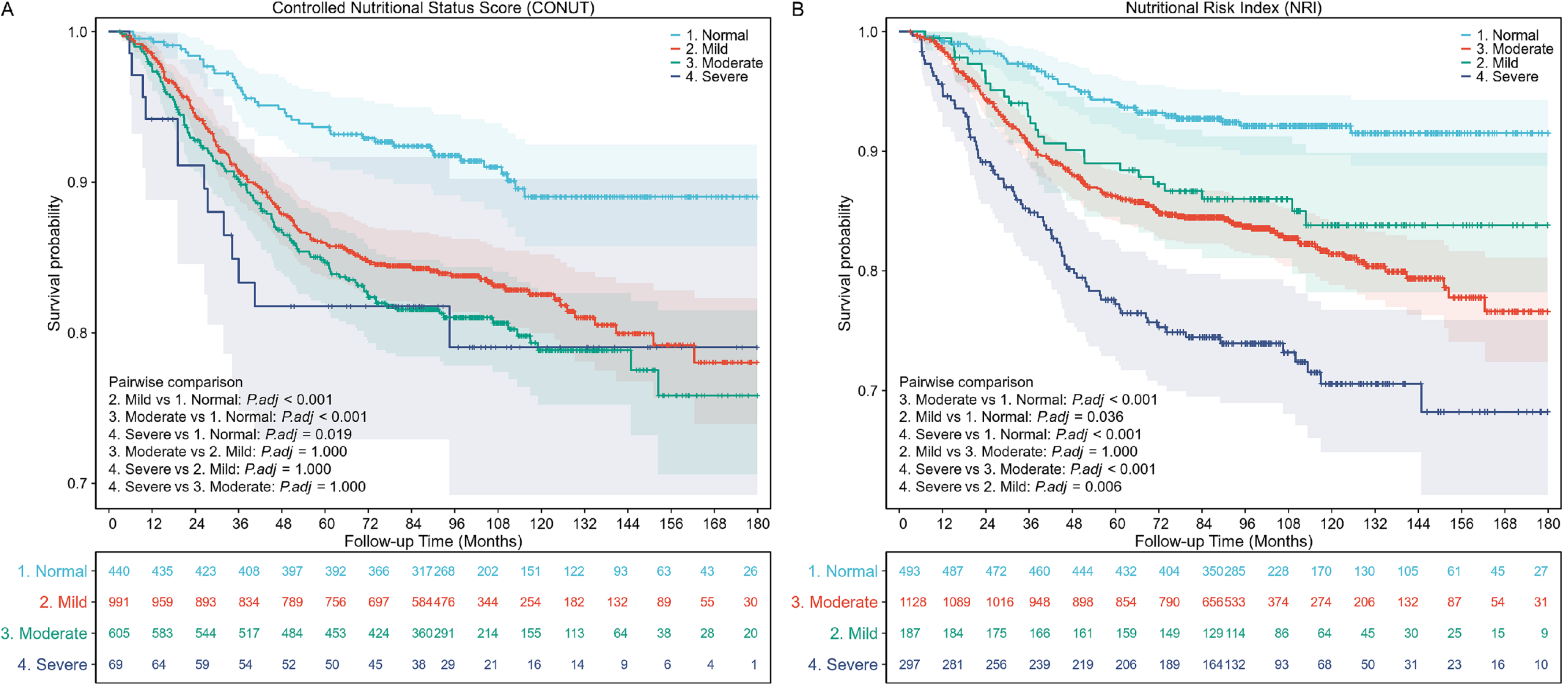
Kaplan–Meier (KM) survival curves stratified by different nutritional risk statuses. **(A)** KM curves according to the Controlled Nutritional Status Score (CONUT) (unweighted). Log-rank test, *p* < 0.001. **(B)** KM curves according to the Nutritional Risk Index (NRI) (unweighted). Log-rank test, *p* < 0.001.

The Kaplan–Meier survival curves indicated significantly higher all-cause mortality in patients with nutritional risk over the 10-year follow-up period, irrespective of whether the CONUT or NRI score was applied (log-rank test, *p* < 0.001; Figure 2).

The Cox proportional hazards regression analysis was conducted to evaluate the impact of nutritional risk on all-cause mortality. The analysis showed that the risk of all-cause mortality increased for each one-point increment in the CONUT score (aHR, 1.07; 95% CI, 1.02–1.12; *p* = 0.008) and decreased for each one-point increment in the NRI score (aHR, 0.97; 95% CI, 0.96–0.98; *p* < 0.001).

In the fully adjusted model (Model 3), compared with patients with normal nutritional status, the adjusted hazard ratio (aHR) for all-cause mortality was 1.87 (95% CI, 1.30–2.67; *p* < 0.001) for patients with mild nutritional risk screened by CONUT, and 1.84 (95% CI, 1.12–3.03; *p* = 0.016) for patients with mild nutritional risk screened by NRI. For severe nutritional risk, the aHRs for all-cause mortality were 2.55 (95% CI, 1.34–4.85; *p* < 0.001) and 2.64 (95% CI, 1.74–4.03; *p* < 0.001) according to CONUT and NRI, respectively (Table 3).

**Table 3:** All-cause mortality hazard ratios (HRs) for patients according to different nutritional risk status.

Risk factor	Model 1		Model 2		Model 3	
	HR (95% CI)	*p* value	HR (95% CI)	*p* value	HR (95% CI)	*p* value
CONUT, continuous						
Per 1-score increment	1.10 (1.05 ~ 1.15)	<0.001	1.08 (1.03 ~ 1.13)	<0.001	1.07 (1.02 ~ 1.12)	0.008
CONUT, categorical						
Normal	Ref					
Mild	2.00 (1.41 ~ 2.82)	<0.001	2.00 (1.41 ~ 2.83)	<0.001	1.87 (1.30 ~ 2.67)	<0.001
Moderate	2.32 (1.62 ~ 3.33)	<0.001	2.17 (1.51 ~ 3.12)	<0.001	1.87 (1.27 ~ 2.74)	0.001
Severe	2.46 (1.32 ~ 4.60)	0.005	2.13 (1.13 ~ 4.00)	0.019	2.55 (1.34 ~ 4.85)	0.004
NRI, continuous						
Per 1-score increment	0.96 (0.95 ~ 0.97)	<0.001	0.97 (0.95 ~ 0.98)	<0.001	0.97 (0.96 ~ 0.98)	<0.001
NRI, categorical						
Normal	Ref					
Mild	1.98 (1.20 ~ 3.25)	0.007	1.93 (1.18 ~ 3.18)	0.009	1.84 (1.12 ~ 3.03)	0.016
Moderate	2.46 (1.73 ~ 3.50)	<0.001	1.94 (1.35 ~ 2.79)	<0.001	1.66 (1.15 ~ 2.39)	0.007
Severe	4.11 (2.78 ~ 6.08)	<0.001	3.25 (2.15 ~ 4.90)	<0.001	2.64 (1.74 ~ 4.03)	<0.001

Model 1: No adjusted. Model 2: Adjusted by age, gender, BMI, hyperlipemia, diabetes, cardiovascular disease. Model 3: Model 2 plus serum uric acid, serum phosphorus, serum potassium, serum alkaline phosphatase, iPTH, FBG, CRP, HGB, Kt/V. CONUT, the Controlled Nutritional Status Score; NRI, the Nutritional Risk Index; BMI, body mass index; iPTH, intact parathyroid hormone; FBG, fasting blood glucose; CRP, C-reactive protein; HGB, hemoglobin; HR, hazard ratio; CI, confidence interval.

**Figure 3:**
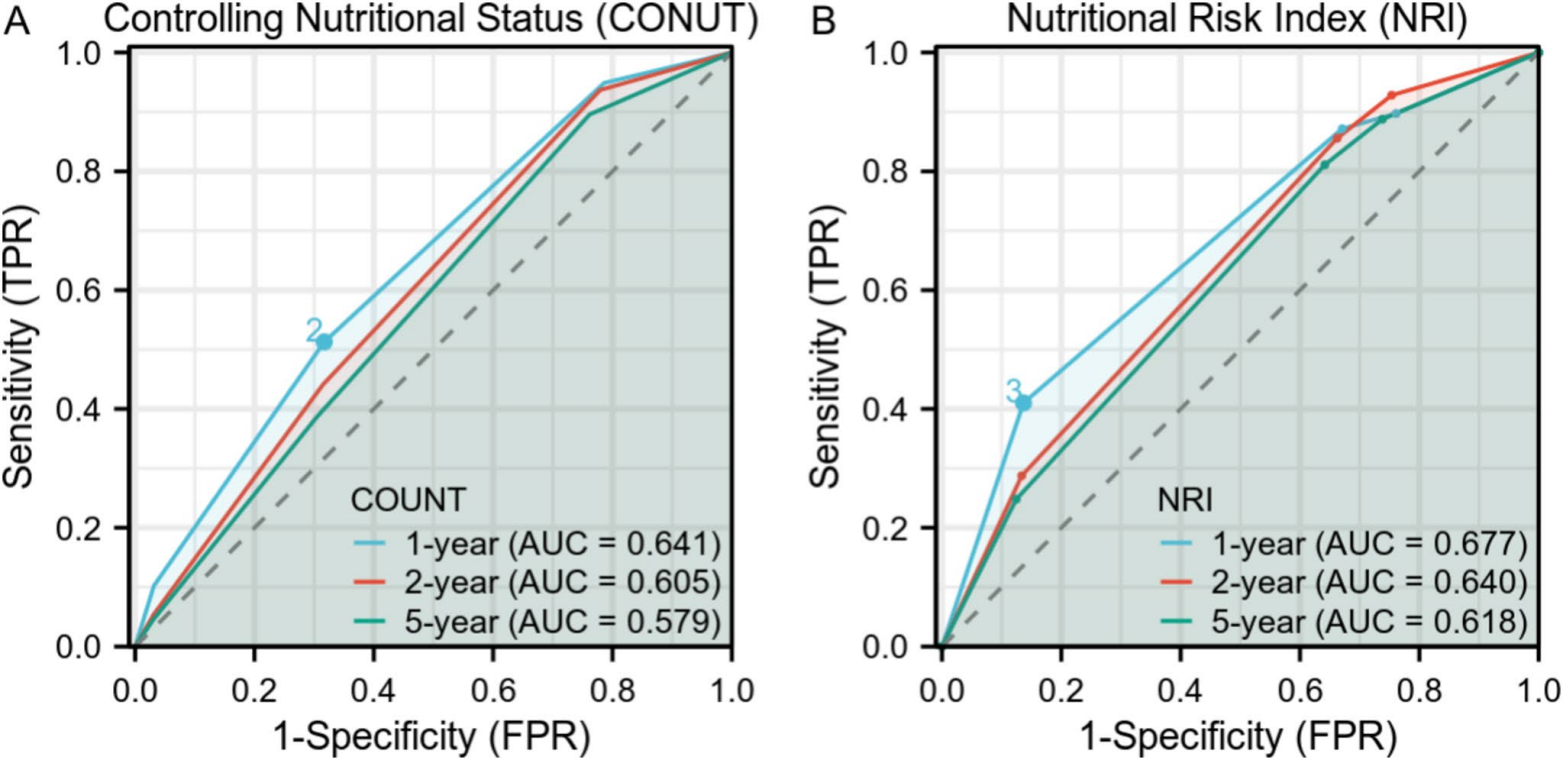
Time-Dependent Receiver Operating Characteristic (ROC) Curves for Controlling Nutritional Status (CONUT) and Nutritional Risk Index (NRI) over 1-year, 2-year, and 5-year intervals. **(A)** ROC curves for CONUT with Area Under Curve (AUC) values of0.641, 0.605, and 0.579 for 1-year, 2-year, and 5-year intervals, respectively. **(B)** ROC curves for NRI with AUC values of 0.677, 0.640, and 0.618 for 1-year, 2-year, and 5-year intervals, respectively.

The Receiver Operating Characteristic (ROC) curves in Figure 3 show the time-dependent diagnostic performance of the Nutritional Risk Index (NRI) and CONUT. For the CONUT dataset (Figure 3A), the Area Under the Curve (AUC) values for 1-year, 2-year, and 5-year are 0.641, 0.605, and 0.579, respectively. For the NRI dataset (Figure 3B), the AUC values were 0.677 for 1-year, 0.640 for 2-year, and 0.618 for 5-year.

### Subgroups analysis

**Figure 4:**
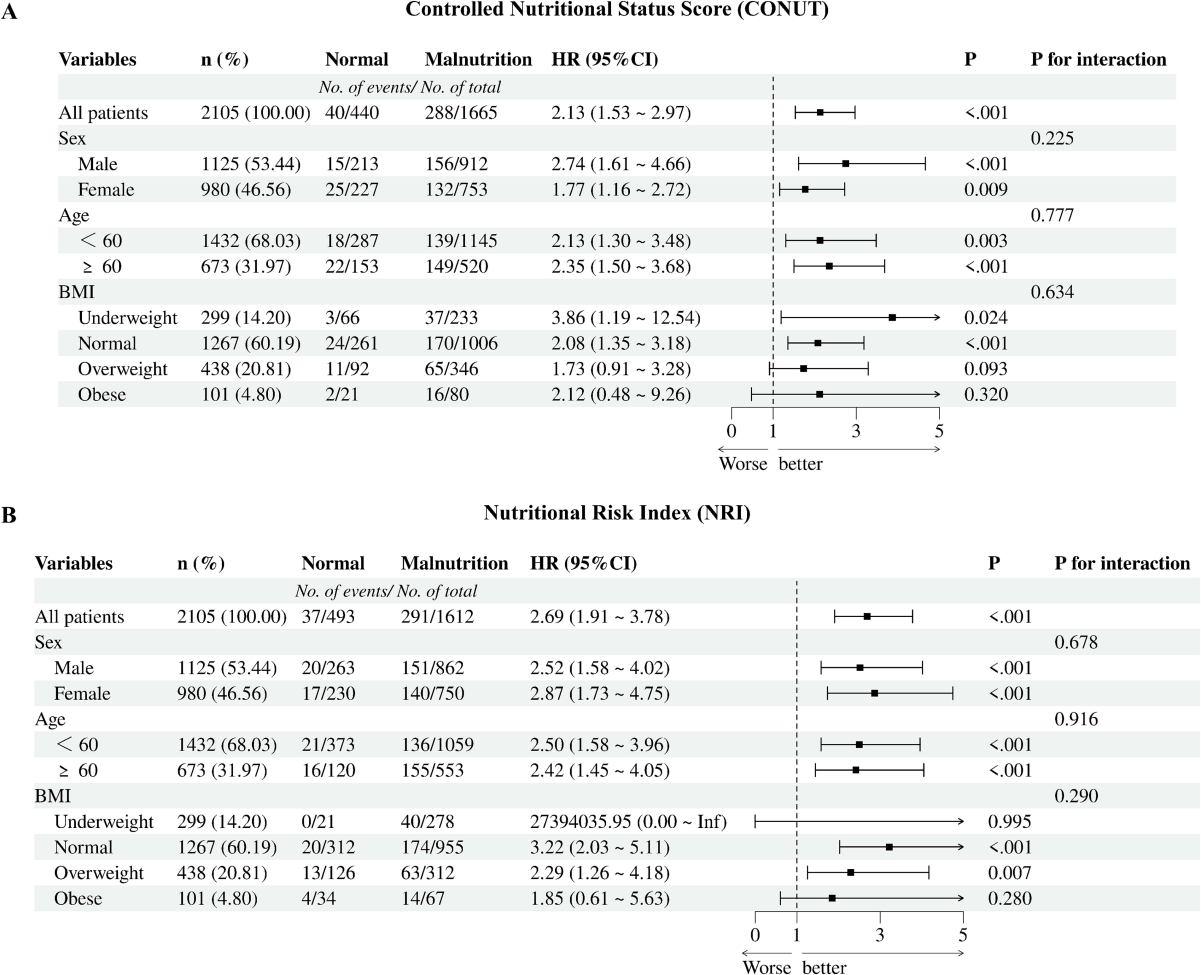
Cox proportional hazards regression models of different malnutrition status and all-cause mortality. **(A)** CONUT, the Controlled Nutritional Status Score; **(B)** NRI, the Nutritional Risk Index; BMI, body mass index.

We performed subgroup analysis in some subgroups that we were interested in and explored the interaction between the subgroups and nutritional risk. The forest plot showed no interaction between the subgroups (Figure 4).

## Discussion

Nutritional risk is a critical issue among patients undergoing peritoneal dialysis (PD), significantly affecting their overall health and quality of life. This condition, often worsened by dialysis-related factors such as protein loss, inadequate intake, and inflammation, leads to adverse outcomes like increased hospitalization and mortality (8–10). Thus, effective and standardized nutritional risk screening protocols are crucial for optimizing patient care (11, 12).

Our multicenter retrospective study with 2,105 PD patients assessed nutritional risk prevalence and its impact on mortality using CONUT and NRI. Both scores revealed a high nutritional risk burden, with 76.58 and 79.10% of patients classified as high-risk by CONUT and NRI, respectively. Severe nutritional risk was independently associated with an increase in mortality hazard (CONUT-adjusted HR = 2.55; NRI-adjusted HR = 2.64). These findings highlight the necessity of integrating regular nutritional screening into PD patient care to improve outcomes (8, 13).

Current approaches to nutritional risk evaluation in PD patients predominantly rely on established frameworks such as the Global Leadership Initiative on Malnutrition (GLIM) and the Subjective Global Assessment (SGA). Notably, the development of GLIM was built upon earlier standards from the European Society for Clinical Nutrition and Metabolism (ESPEN) and the American Society for Parenteral and Enteral Nutrition (ASPEN). The ASPEN 2012 consensus established a comprehensive framework integrating both subjective assessments (e.g., Subjective Global Assessment) and objective tools (e.g., Malnutrition Universal Screening Tool) to standardize malnutrition diagnosis in hospitalized populations, emphasizing multidisciplinary collaboration and proactive nutritional interventions (14). Similarly, the ESPEN 2015 guidelines introduced multidimensional evaluations, combining anthropometric measurements, biochemical markers, and clinical assessments to enhance diagnostic accuracy, particularly in chronic disease and surgical patients (15). The GLIM criteria, formalized through a consensus by major societies including ESPEN and ASPEN, provide a two-step diagnostic framework: phenotypic criteria (e.g., weight loss >5%, low body mass index [BMI] < 18.5 kg/m^2^, or reduced muscle mass—a hallmark of sarcopenia) and etiologic criteria (e.g., reduced dietary intake or chronic inflammation) (1). This framework explicitly recognizes sarcopenia-related muscle depletion as a central component of malnutrition risk stratification. In contrast, SGA, as the most widely used clinical tool, relies on subjective clinician assessments of weight changes, dietary intake, and physical signs of muscle or fat wasting (16, 17).

The simplicity of SGA allows for quick bedside evaluations but its reliance on clinician judgment can limit its sensitivity in fluid-overloaded PD patients, where fluid retention might be mistaken for improved nutrition (18). Advanced techniques like bioelectrical impedance analysis (BIA) can more accurately assess dry body weight by distinguishing fluid from lean and fat mass, providing clearer insights into nutritional status (19). BIA is crucial for early detection of sarcopenia in PD patients, where fluid can mask muscle loss, allowing for timely nutritional interventions to preserve muscle health (20). This method provides a more precise evaluation by differentiating between the water weight and actual body composition, offering a clearer insight into the patient’s nutritional state (21).

Although these diagnostic frameworks are methodologically thorough, their implementation often requires specialized resources (e.g., imaging for muscle mass quantification, multidisciplinary coordination), which challenges routine use in PD practice. In this context, CONUT and NRI offer a streamlined alternative for rapid screening. The CONUT score integrates three routinely measured parameters—serum albumin, lymphocyte count, and total cholesterol—to generate a risk score ranging from 0 to 12 (7). The NRI calculates risk using current weight/ideal weight and albumin levels (6). Both tools enable efficient risk stratification, with CONUT identifying 67.7% of patients with moderate-to-severe risk and NRI classifying 75.82% as mild-to-moderate risk. These differences highlight their complementary roles: CONUT’s emphasis on inflammation may prioritize patients needing anti-inflammatory interventions, while NRI’s focus on energy-protein balance could guide dietary support.

ROC curve analysis provides insights into how well NRI and CONUT perform as diagnostic tools for predicting mortality in PD patients over different time periods. Although AUC values suggest an acceptable but lower predictive ability, NRI generally shows slightly better. The decline in AUC values over time highlights challenges in long-term prognostication. Our findings suggest a two-step nutritional care pathway: first, CONUT/NRI can assist in screening to identify high-risk patients, followed by a confirmatory evaluation using GLIM criteria to diagnose and grade malnutrition.

Study limitations include reliance on retrospective data, which may introduce biases and limit causality. Our sample, though substantial, is from multiple centers within a single country, affecting external validity. Additionally, the lack of clinical validation raises concerns about applicability in practice, necessitating future prospective studies incorporating diverse demographics and rigorous validation.

In conclusion, this study suggests that the Controlling Nutritional Status (CONUT) score and the Nutritional Risk Index (NRI) can assist in screening for nutritional risk in PD populations. Their ability to capture both inflammatory and catabolic pathways enables targeted interventions that address multifactorial nutritional deterioration. While these tools incorporate into PD care protocols represent an important step towards reducing morbidity. Future studies should focus on validating dynamic monitoring approaches, including the frequency of re-evaluation and the integration of additional parameters, to enhance the accuracy and comprehensiveness of nutritional assessments in this population.

## Data Availability

The raw data supporting the conclusions of this article will be made available by the authors, without undue reservation.
